# The shapes of brain waste: Mysteries of cellular remnant morphology in neurodegeneration

**DOI:** 10.17879/freeneuropathology-2026-9059

**Published:** 2026-01-21

**Authors:** David V. Forrest

**Affiliations:** 1 Columbia University Vagelos College of Physicians & Surgeons, Rye, New York, USA

**Keywords:** Community, Gestalt, Histology, Inclusions, Laypersons, Neurodegeneration

## Abstract

This meditation with apologies by a psychiatrist who is not a neuropathologist but attends CPCs of movement disorder patients speculates about how microscopic mechanical processes produce the variety of cellular neuropathological forms, which differ with diagnoses, and wonders what may be revealed by the shapes of these traces by future neuropathologists. New discoveries about tiny machines, nanotubules and the like only increase the intrigue and possibilities of revelations, likened to a crime scene investigation.

## CONTACT

CONTACT (Community-Oriented Neuropathology Thoughts And Clarification Threads) facilitates interaction between neuropathology and the public. See the response paper: https://doi.org/10.17879/freeneuropathology-2026-9241

## CONTACT

This meditation about neuropathology by a psychiatrist was stimulated by a talk given by Hasini Reddy, M.D., our neuropathologist at the Movement Disorder Group at Columbia University, on August 6, 2024. After her comprehensive taxonomy of the varieties of neurodegenerative cells in various disorders, I sent her the following speculations and asked her if neuropathologists could explain why the dead cells take the various forms that they do. In her reply on August 20, 2024 (1), she thanked me for my “very interesting musings” which she found “poetic and thought-provoking”.

Aside from medical school basics, and my attendance at clinical-pathological conferences, I have no background in neuropathology and thus speculate without much help or hindrance from knowledge. I was emboldened because in psychiatry we welcome the reactions of those outside our field, and find them by turns challenging, interesting, informative, heuristic, or amusing.

So, I began to imagine what this menagerie of damaged and dead little creatures might say to us if they themselves could speak about what had happened to them and had led to their demise:

“We are the corpses of cortical cells, neurons and astros and oligos. We haunt the microscopic field as ghosts, and we suggest the potential of more to be revealed right under the nose. We look very different now from when we were alive and healthy, and before we swirl down the glymphatic drain. Some of us form snarled plaques. Some of us in spongiosis are vacuolated to emptiness. We who are astrocytes become tufted around a dense center. Others look horned or as if we had an unruly bad hair day or have exploded. Some of us who are neurons in corticobasal degeneration balloon to a globular girth. Some of us wear silly Smurf hats. Little halos as if imitating microsainthood abound. Others harbor sleek tumescent spheres that indent or push the nucleus aside. Sinister coiled bodies snake about in progressive supranuclear palsy and corticobasal degeneration. Plentiful threads that once were dendrites are strewn around like cables on the floor of a television studio or downed power lines after a storm. In short, it often looks like the aftermath of microscopic violence. The proportionately incredible length alone of neurite processes such as axons makes waste removal in neuropathology an insurmountable physical challenge for intracellular transport. It suggests the possibility of shifting the focus of studying us from chemistry to nanomechanics. Much is known about our genetics and chemistry, the synuclein or tau proteins we are made of, and their contiguous, spreading misfolding. But the mystery remains of how in dying we come to take the shapes that we do. What are the several ways that lead to pathological shapes? Starvation? Competitive elimination? Asphyxiation? Intoxication, auto- or allo-? Explosion by expanding tiny bubbles of bad gas or liquid? What’s the gas? Buildup of ingested or unexcreted waste? Call in Detective Dick Tracy from the Sunday newspaper comic strip which assumed facial peculiarities indicated iniquity, or television’s *Crime Scene Investigation*. In the board game *Clue*, was it Colonel Mustard in the kitchen? There may be value in wringing such information from us.”

It would not be surprising if there was significance in the shapes of cellular detritus. Michio Kaku (*Quantum Supremacy*, Vintage, 2023, p. 187) (2) notes that in the growing understanding of proteinomics, “function follows form” in that it is the shape of a protein molecule (rather than its chemical composition) that produces protein function. Thus, the field of “the protein folding problem”, mapping protein shape, may be the key to diseases they produce (as well as all of their effects).

Let us pause for a moment and consider the strangeness of this. An analogy might be that a committee assembling a team of biomedical scientists for a specific task would choose them by a Miss America bathing suit contest, or a Mr. America muscle building contest, rather than the scientists’ proven mental abilities and accomplishments.

But then, if we think again, it is not so strange when we consider that evolution, the great computational problem solver over the eons, has availed itself of every possible manner of difference in natural selection from random mutations. Granted that the component atomic structures contribute to molecular profiles, why would nature *not* avail itself of external shape? A key that opens a lock can be made of silver or brass or lead, and nature has never been constrained by appearances. Not only that, but experiments have found kindergarteners believe prettier teachers are smarter, and Dick Tracy’s villains had observable deformities supposedly betraying inner evil. These assumptions may be false but are popular prejudices.

Taking this further, every step of dismantling the intricate structure of cellular machinery poses disposal problems of the shapes of brain garbage. When the junk luggers arrive in the brain, they need to equip themselves with tools and procedures specific for those shapes. In a city or the heavily built-up suburb in which I live, it may be expensive to tear down a house, over 20% of the cost to build one. One cannot simply blow it up (which also usually takes experts), like the Manhattan townhouse an angry divorced man prevented his wife from getting, thereby killing himself and granting her a more valuable buildable lot (Gay Talese, *Bartleby and Me*, Mariner Books, 2023) (3).

Barrel-shaped molecular complexes called chaperones are now known to be involved in *proteostasis*, or the maintenance of proteins in their functioning folded state, and mutations that impair the chaperones may cause brain disorders called “chaperonopathies” (Manu Sharma, “Some brain disorders are ‘chaperonopathies’”, *Science*, 2024, 386 pp. 496–7) (4). Why wouldn’t they?

A stereoscopic knowledge of our miniature world has progressed, from the basics of DNA and RNA structure by crystallography to the fitting shapes of antibodies.

Evidence grows that the environment of this world is not a general chemical soup in which ingredients find one another by random encounters, but a highly structured factory in which siloed, sorted and meaningfully rearranged elements are assembled in corridors by tiny machines as ingenious as nature can devise.

But how the polymorphic shapes of detritus are formed in neurodegeneration is yet to be explained.

Actions in the microscopic world don’t just happen, as if shaken or stirred into place. Molecular-level machines do mechanical work. Nothing moves without forces exerted or tiny engines operating. Cells divide with little strings attached. Cells are amazingly mobile and agile beyond simple tropisms and osmosis in a jostling environment of Brownian weather. In our bodies the motility devices of precursor animals survive, ciliated and flagellated, or pseudopoded. On closer look, the web of mitochondria acts like a Congressional budget office doling out dollops of energy grants by unknown processes and may contribute an intelligence of its own dating from our primeval mothers.

The mitotic spindle is a complex cytoskeletal machine with several moving parts and microtubules that pull apart the replicated daughter chromosomes apart toward poles they are also moving apart. Is the exploded architecture of some defunct astros the result of a tiny centrifugal push or just the dying off of central structures? Do Lewy bodies and other globular elements contract centripetally? Does a molecular membrane contain them? Are they electrostatically bounded with surface tension like raindrops and soda bubbles? How do Lewy bodies push on and indent a nuclear membrane? What propels them to displace? Are there traction and tensile pulls, molecular motors? As structures like the network of dendritic nanotubules are characterized (Dimitri Budinger and Michael T. Heneka, “Hidden networks in the brain”, *Science*, 2025, 390 pp. 25–26) (5), more questions arise about how they are constructed, work and are maintained. Who belong to the nanoplumbers’ union and how do they apprentice to fix nanoleaks?

Do some cell remnants remain longer? Is some trash not picked up or drained so quickly as other trash? Is some never ingested and carried away by macrophages, like a garbage strike in the Bronx? Are some terminally entombed? Kept in a vase on the mantel, as it were?

There are wonders, perhaps useful, to be learned from refuse for a future Malpighi, Purkinje, Cajal or Golgi to discover.

Nanorobots are on their way in many branches of science, perhaps most in biomedicine. This branch of innovation is developing nanoscopic robotic tools that will be able to operate within the body to manipulate such objects as our neuropathic garbage, when we are able to “swallow the doctor.”

Farther in the future is understanding of the deeper quantum level already being built in computation, the promise of which is explained by Michio Kaku in *Quantum Supremacy: How The Computer Revolution Will Change Everything* (Vintage, 2023) (2): “Alzheimer’s, Parkinson’s and ALS [amyotrophic lateral sclerosis] … do their damage at the molecular level, which only quantum computers can unravel and help fight” (p. 160).

I hope that these loose and as yet unsupported speculations may inspire new workers with new tools to achieve new understanding.

**Figure 1:**
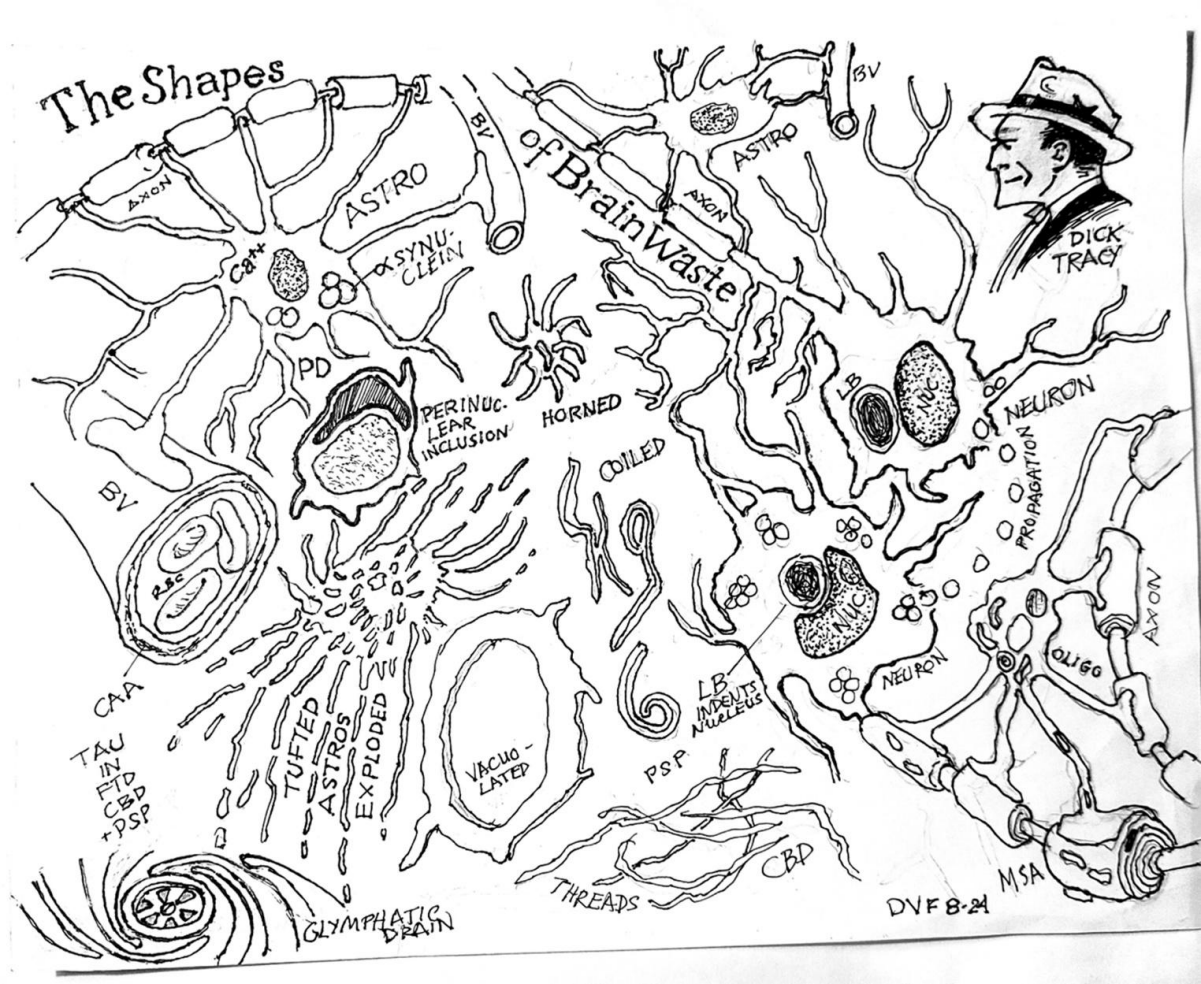
Key: **BV** = blood vessel, **Astro** = astrocyte, **RBC** = red blood cell, **CAA** = cerebral amyloid? angiopathy, **PD** = Parkinson’s disease, **Tau** = tau protein, **FTD** = frontotemporal degeneration, **CBD** = corticobasal degeneration, **PSP** = progressive supranuclear palsy, **LB** = Lewy body, **Oligo** = oligodendrocyte, **MSA** = multiple system atrophy, **Dick Tracy** = comic strip detective from the past.

Note: The torpedo axonal swellings and dendritic swellings of Purkinje cells under stress in the cerebellum being studied by Elan Louis, Sheng-Han Kuo and Phyllis Faust (NINY Neurological Institute of New York? Chairman’s Desk 14:37, 2024) warrant special attention. How might the formation of pathology be? related to the morphology of Purkinje cell axons, which are shorter than cortical neurons with early lateral branching?

Finally, a disclaimer: The author is a psychiatrist who consults to the Movement Disorder Group at Columbia University’s Neurological Institute and regularly attend clinical-pathological conferences in which the gross and microscopic pathology is presented. These remarks are not otherwise constrained by knowledge and experience, and so indulge in free speculation. Rather than presuming to contribute to a complex field in which stereochemical shapes are a part, I propose an exploration of the material science and physical engineering in the deconstruction of these microscopic structures.

## Conflict of interest statement

The author declares no conflict of interest.

## References

[R1] Reddy H: Personal communication, 20 Aug 2024.

[R2] Kaku M: Quantum Supremacy: How The Computer Revolution Will Change Everything, Vintage, 2023, pp. 160, 187.

[R3] Talese G: Bartleby and Me: Reflections of an Old Scrivener, Mainer Books, 2023.

[R4] Sharma M: Some brain disorders are ‘chaperonopathies:’ Mutations that impair a protein-folding chaperone can lead to brain malformations, Science 2024, 386 pp. 496–7. 10.1126/science.adt0039 39480954

[R5] Budinger D and Heneka MT: Hidden networks in the brain: Dendritic nanotubes extend brain connectivity beyond synapses, Science 2025, 390 pp. 25–26. 10.1126/science.aeb2962 41037630

